# Extract of* Rhus verniciflua* Stokes Induces p53-Mediated Apoptosis in MCF-7 Breast Cancer Cells

**DOI:** 10.1155/2019/9407340

**Published:** 2019-02-07

**Authors:** Min Sung Kim, Chul Won Lee, Jung-Hoon Kim, Jang-Cheon Lee, Won Gun An

**Affiliations:** ^1^School of Korean Medicine, Pusan National University, Yangsan 50612, Republic of Korea; ^2^Longevity life Science and Technology Institutes, Pusan National University, Busan 46241, Republic of Korea; ^3^Research Institute for Korean Medicine, Pusan National University, Yangsan 50612, Republic of Korea

## Abstract

*Rhus verniciflua* Stokes has long been used as a food supplement and traditional herbal medicine for various ailments in East Asia. We evaluated the anticancer effects of* Rhus verniciflua* Stokes extract (RVSE) on MCF-7 cells by 3-(4,5-dimethylthiazol-2-yl)-2,5-diphenyltetrazolium bromide assay, flow cytometry, annexin V/7-AAD staining, and western blotting. In addition, the gallic acid content of RVSE was assayed using high-performance liquid chromatography. RVSE inhibited the growth of MCF-7 cells in a dose-dependent manner by inducing apoptosis in the sub-G1 phase. RVSE also significantly increased the number of apoptotic cells and increased the expression of p53 and p21 in a dose-dependent manner. Furthermore, RVSE treatment increased the Bax:Bcl-2 ratio and the levels of apoptosis-related factors, such as cleaved caspase-3 and -9 and PARP, in MCF-7 cells. Our findings suggest that the proapoptotic effect of RVSE on MCF-7 cells is mediated by p53, p21, and the intrinsic mitochondrial cascade. Thus, RVSE shows promise for the prevention and treatment of breast cancer.

## 1. Introduction

Cancer is characterized by the uncontrolled growth and spread of abnormal cells and is caused by hormones, immune conditions, and deregulation of oncogenes [1]. Deregulation of oncogenes results in explosive cell proliferation and induction of invasiveness, which promotes the acquisition of a malignant phenotype [2]. Breast cancer is one of the most common malignancies in women worldwide. Nearly 200,000 women are diagnosed with invasive breast cancer and around 40,000 die annually; thus, breast cancer is the second leading cause of cancer-related deaths in women globally [1]. The estrogen receptor-positive MCF-7 breast cancer cell line was derived from the pleural effusion of a patient with metastatic breast cancer [3]. Several decades of use have facilitated the evolution of distinct MCF-7 lineages resistant to chemotherapy [3, 4].

The p53 gene encompasses 16–20 kb of DNA on the short arm of human chromosome 17 and is involved in prostate, lung, colorectal, and breast carcinogenesis [5, 6]. In normal cells, p53 is present at a low concentration and its production is tightly regulated [7]. However, mild stress induces a slight alteration in the p53 level, resulting in transient cell cycle arrest to allow repair of damaged DNA, whereas severe stress and possibly irreparable DNA damage lead to an increase in the p53 level, followed by apoptosis [8–10].

*Rhus verniciflua* Stokes, a deciduous tree of the Anacardiaceae family, has long been used as a food supplement and traditional herbal medicine for various ailments in East Asia [11].* R. verniciflua* Stokes extract (RVSE) reportedly exerts antimicrobial [12], antimutagenic [13], antiarthritic [14], antiplatelet [15], antioxidant [16], anti-inflammatory [17], and anticancer [18–21] effects. However, the p53-dependent mechanism of the induction of apoptosis in breast cancer cells by RVSE is unclear. Therefore, we investigated the antiproliferative and antiapoptotic effects of RVSE in MCF-7 cells. RVSE-induced apoptosis by upregulating the p53 level in a dose- and time-dependent manner and by activating apoptosis-associated proteins, such as Bax/Bcl-2, cleaved caspase-3 and -9 and cleaved PARP, in MCF-7 cells.

## 2. Materials and Methods

### 2.1. Materials

Dulbecco's modified Eagle's medium (DMEM), fetal bovine serum (FBS), and Dulbecco's phosphate-buffered saline (D-PBS) were purchased from Gibco (Grand Island, NY, USA). Monoclonal antibodies specific for Bax, Bcl-2, cleaved caspase-3 and -9, cleaved PARP, and PARP were obtained from Sigma-Aldrich (St. Louis, MO, USA). Anti-p53 and -*β*-actin antibodies were purchased from Santa Cruz Biotechnology (Santa Cruz, CA, USA). Gallic acid was purchased from ChemFace (purity ≥98%; Wuhan, Hubei, China). Acetonitrile, methanol, and water (HPLC-grade) were obtained from JT Baker Inc. (Phillipsburg, NJ, USA). Trifluoroacetic acid, 3-(4,5-dimethylthiazol-2-yl)-2,5-diphenyltetrazolium bromide (MTT), and other reagents were obtained from Sigma-Aldrich.

### 2.2. Preparation of RVSE* R. verniciflua*

Stokes (Anacardiaceae) was purchased from Hwalim Natural Drug Co., Ltd. (Busan, South Korea) and was identified by Prof. Jang-Cheon Lee (School of Korean Medicine, Pusan National University, Yangsan, South Korea). A voucher specimen (PNU15-7) was deposited in the School of Korean Medicine, Pusan National University.* Rhus verniciflua* Stokes (25 g) was placed into a fivefold volume of ultrapure water and boiled using a heating mantle (Misung, Daejeon, Korea) equipped with a reflux condenser. After 2 h, the suspended solution was twice passed through a hydrophilic polytetrafluoroethylene filter (Advantec, Tokyo, Japan) and concentrated in a rotary evaporator (Eyela, Tokyo, Japan) at 40°C, lyophilized (Labocnco, MO, USA) for 24 h, and stored at −20°C. The yield of lyophilized RVSE was 10.3%. Samples were dissolved in distilled water and passed through a 0.25-*μ*m syringe filter (Sigma-Aldrich) before use.

### 2.3. Chemical Profiling of RVSE by High-Performance Liquid Chromatography

#### 2.3.1. Chromatography Conditions

HPLC was performed using an Agilent 1200 instrument (Agilent Technologies, Palo Alto, CA, USA) equipped with a quaternary pump, autosampler, column oven, and diode-array detector. Data were acquired by ChemStation software (rev. B.03.02; Agilent). Chromatographic separation was performed on a Capcell Pak Mg II C_18_ column (4.6 × 250 mm, 5 *μ*m; Shiseido, Tokyo, Japan) at 30°C. The mobile phase consisted of water containing 0.1% trifluoroacetic acid (A) and acetonitrile (B) with the following gradient program: 10% (B) maintained for 1 min, 10–80% (B) over 1–25 min, and reequilibration to 10% (B). The flow rate was set at 1.0 mL/min and the injection volume was 10 *μ*L. The detection wavelength was set at 260 nm.

#### 2.3.2. Preparation of Standard and Sample Solutions

Accurately weighed standard compounds were dissolved in methanol and diluted as appropriate. RVSE was dissolved in methanol, filtered through a 0.2 *μ*m syringe filter (BioFACT™, Korea), and injected into the HPLC instrument.

### 2.4. Cell Lines and Culture

Human breast cancer MCF-7 cells (American Type Culture Collection, Manassas, VA, USA) were cultured in DMEM containing 10% (v/v) FBS and 1% (v/v) penicillin-streptomycin (Gibco) at 37°C in a 5% CO_2_ incubator. Cells were cultured by enzymatic digestion with trypsin-EDTA (0.25% trypsin, 1 mM EDTA) solution upon reaching ~ 80% confluency.

### 2.5. Cell Proliferation Assay

Cell viability was determined by MTT colorimetric assay. MCF-7 cells were seeded in a 96-well plate at a density of 1 × 10^4^ per well. After incubation for 24 h, the cells were treated with 200–400 *μ*g/mL RVSE for 24 h at 37°C in an incubator with a 5% CO_2_ atmosphere. Next, 10 *μ*L of MTT (0.5 mg/mL) in culture medium were added and the plates were incubated for 3 h at 37°C. The medium was aspirated, and the formazan crystals were dissolved in 200 *μ*L of dimethyl sulfoxide (DMSO); the absorption at 570 nm of the resulting solution was read using a microplate reader (Tecan Group Ltd., Männedorf, Switzerland). Data are presented as percentage viabilities of untreated cells (100%). The assay was repeated three times independently.

### 2.6. Flow Cytometry Analysis of the Cell Cycle

MCF-7 cells were seeded into 100 mm dishes. After exposure to RVSE for 24 h, the cells were fixed in 70% ethanol. The cells were next resuspended, incubated with RNase (250 *μ*g/mL final concentration) for 30 min, and stained with propidium iodide (10 *μ*g/mL final concentration) for 1 h. Flow cytometry was performed on a FACS instrument (BD FACSCanto II, BD Biosciences, San Jose, CA, USA) equipped with CellQuestPro software.

### 2.7. Quantification of Apoptosis by Annexin V Labeling

Apoptosis was examined using an Annexin V & Dead Cell Kit (Millipore, Hayward, CA, USA) according to the manufacturer's instructions. Briefly, 1 × 10^6^ MCF-7 cells were seeded in a 60-mm cell culture plate and treated with various concentrations of RVSE for 24 h. The cells were collected and incubated with annexin V and 7-AAD for 20 min at room temperature in the dark. The events for live, dead, early (annexin-V^+^/7-AAD^−^), and late apoptotic (annexin-V^+^/7-AAD^+^) cells were counted using the Muse Cell Analyzer (Millipore).

### 2.8. Western Blot Analysis

Sample preparation and western blot analysis were performed. Briefly, MCF-7 cells were harvested by centrifugation at 4°C, washed three times in ice-cold D-PBS, resuspended in lysis buffer (50 mM Tris [pH 7.5], 2 mM EDTA, 100 mM NaCl, 1% NP-40) containing protease inhibitor cocktail (Sigma-Aldrich), and incubated for 15 min at 4°C. The cell lysates were centrifuged for 15 min at 13,000 rpm at 4°C. The protein concentration of the lysates was determined using a bicinchoninic acid (BCA) Protein Assay Kit (Thermo Scientific) and the lysates were adjusted with lysis buffer. Total proteins (30 *μ*g) were separated in 8–15% sodium dodecyl sulfate-polyacrylamide gels and electroblotted onto nitrocellulose membranes (Thermo Scientific), which were incubated overnight in blocking solution (5% skim milk) at 4°C, followed by incubation with primary antibody for 2 h. The blots were washed three times with Tween 20/Tris-buffered saline (TTBS), incubated with the appropriate horseradish peroxidase-conjugated secondary antibody (1:1,000) for 1 h at room temperature, and washed three times with TTBS. Immunoreactive bands were developed using the Enhanced Chemiluminescence Detection System (ECL Plus, Thermo Scientific). Band density was quantitated using ImageJ software.

### 2.9. Statistical Analysis

Statistical analysis was performed using SPSS software version 23 (IBM Corp., Armonk, NY, USA). Data are presented as means ± standard deviation (SD) of triplicate determinations. Differences in means between groups were subjected to one-way analysis of variance (ANOVA) followed by the least-significant differences (LSD) multicomparison test and independent* t*-test.* P* values < 0.05 were considered indicative of significant differences.

## 3. Results

### 3.1. Analysis of RVSE Composition

**Figure 1:**
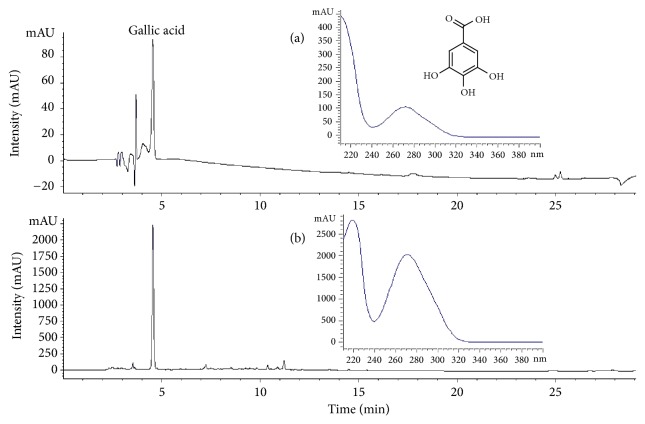
Chromatogram of the major compound identified in* Rhus verniciflua* Stokes extract (RVSE). (a) Chromatogram of the commercial standard compound. (b) Chromatogram of the major compound in RVSE. The chromatograms were obtained at 260 nm.

Gallic acid was identified in the RVSE chromatogram by comparison with the retention time (9.0 min) and UV spectrum of the standard solution. The concentration of gallic acid was calculated using a calibration curve of the standard (124.97 ± 5.30 ppm; Figures 1(a) and 1(b)).

### 3.2. Effects of RVSE on MCF-7 Cell Proliferation and Cell Cycle Arrest

**Figure 2:**
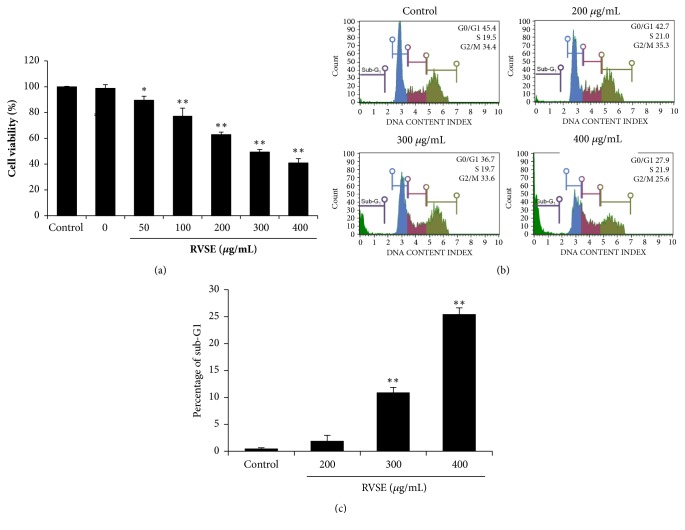
Effects of RVSE on proliferation and cell cycle arrest in MCF-7 cells. (a) Cytotoxicity of RVSE on MCF-7 cells (1 × 10^4^/well) by MTT assay. (b) Cells (5 × 10^5^/mL) were treated with 200–400 *μ*g/mL RVSE for 24 h, fixed, stained with PI, and analyzed by flow cytometry. (c) Percentage of cells in sub-G1 phase. Data are means ± SD of three independent experiments. *∗P* < 0.05 and *∗∗P* < 0.01, significant difference from the control.

To examine its anticancer activity, we assessed the effects of 0–400 *μ*g/mL RVSE on the proliferation of MCF-7 cells (Figure 2(a)). RVSE significantly inhibited the proliferation of MCF-7 cells in a dose-dependent manner (*P* < 0.05 and* P* < 0.01). We also investigated the effect of RVSE on cell cycle arrest. MCF-7 cells were treated with 200, 300, or 400 *μ*g/mL RVSE for 24 h and subjected to cell cycle analysis via flow cytometry. The percentage of sub-G_1_ phase (apoptotic) cells was significantly increased by treatment with 300 and 400 *μ*g/mL RVSE in a dose-dependent manner (*P* < 0.01). Moreover, 400 *μ*g/mL RVSE increased the proportion of cells in sub-G_1_ phase to 25.47% (Figures 2(b) and 2(c)).

### 3.3. Induction of Apoptosis in MCF-7 Cells by RVSE

**Figure 3:**
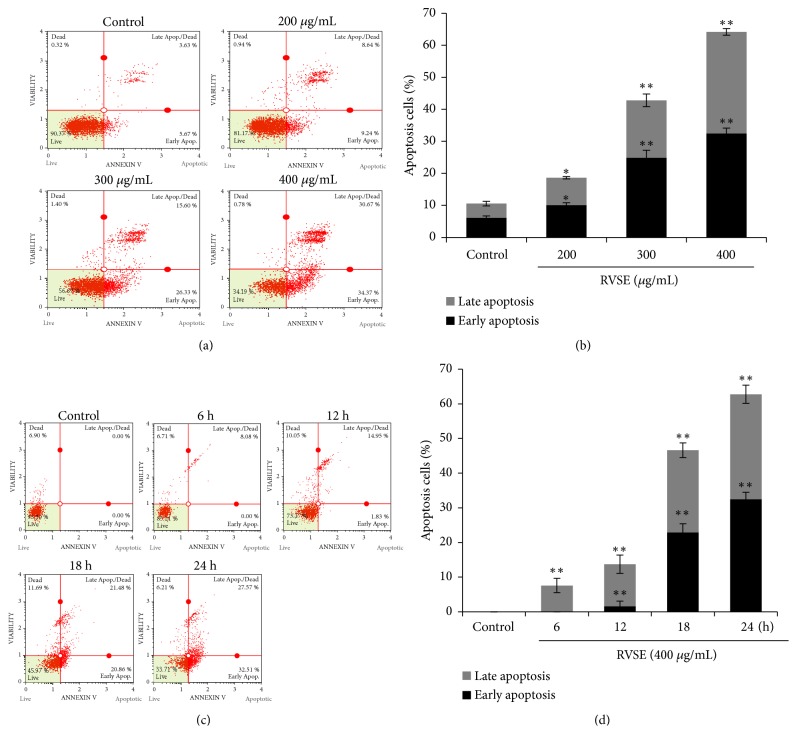
RVSE-induced apoptosis of MCF-7 cells. (a) and (c) Flow cytometric analysis of RVSE-induced apoptosis of MCF-7 cells. (b) and (d) Statistical analysis of (a) and (c). Data are means ± SD of three independent experiments. *∗P* < 0.05 and *∗∗P* < 0.01, significant difference from the control.

To determine whether the RVSE-mediated reduction in the viability of MCF-7 cells was due to apoptosis, we conducted annexin V and 7-AAD staining. The results confirmed the induction of apoptosis (Figure 3). Treatment with RVSE resulted in the presence of viable (annexin V^−^/7-AAD^−^), early apoptotic (Annexin V^+^/7-AAD^−^), late apoptotic (Annexin V^+^/7-AAD^+^), and necrotic (Annexin V^−^/7-AAD^+^) cells. In the control, 10.57% of MCF-7 cells were apoptotic, whereas 18.61, 42.81, and 64.19% of the cells treated with 200, 300, and 400 *μ*g/mL RVSE, respectively, were apoptotic; this represents a dose-dependent effect (*P* < 0.05 for 200 *μ*g/mL;* P* < 0.01 for 300 and 400 *μ*g/mL; Figures 3(a) and 3(b)). In addition, the effect of RVSE (400 *μ*g/mL) on the time (0–24 h) was determined. There were significant increases to the proportion of apoptotic cells at increasing time of RVSE treatment (*P *< 0.01 for 6–24 h vs. Control, Figures 3(c) and 3(d)).

### 3.4. Effect of RVSE on the p53 and p21

**Figure 4:**
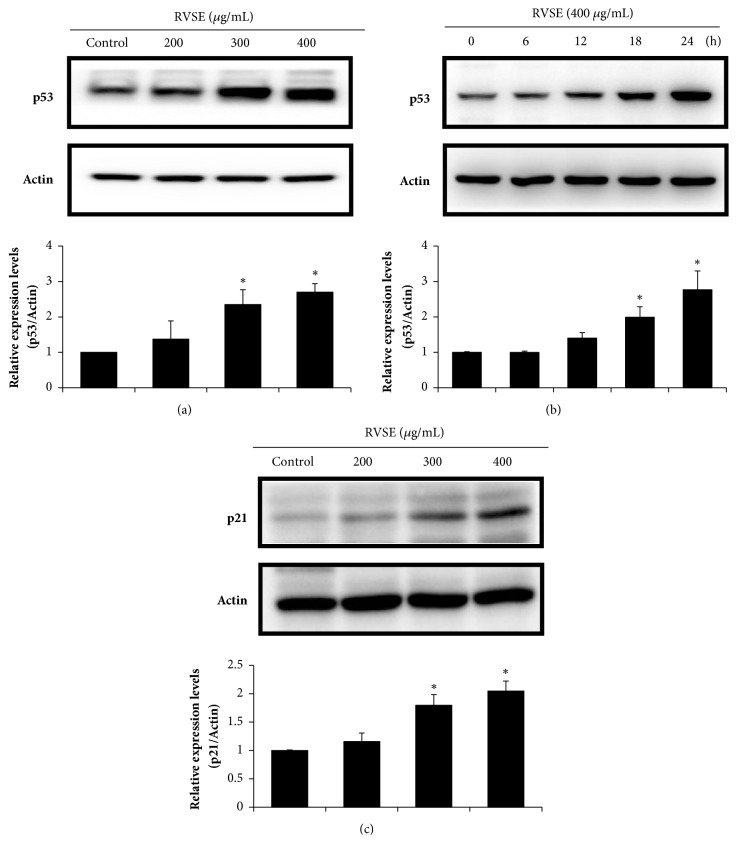
Effect of RVSE on p53 and p21. (a) and (c) Cells (5 × 10^5^/mL) were treated with 200, 300, or 400 *μ*g/mL RVSE for 24 h. (b) p53 levels (time: 0–24 h) in the lysate of cells treated with 400 *μ*g/mL RVSE were determined. Western blot analysis of the (a) and (b) p53 and (c) p21 protein level; *β*-Actin was used as the loading control. A representative of three blots yielding similar results is shown. Relative levels (protein* vs.β*-actin) were determined by densitometry. Data are means ± SD of three independent experiments. *∗P* < 0.05, significant difference from the control.

The effect of RVSE on the p53 and p21 in the doses (200, 300, and 400 *μ*g/mL) and time (0–24 h) was determined by western blotting. RVSE increased the p53 level in a dose- and time-dependent manner (*P* < 0.05 for 300 and 400 *μ*g/mL, and 18 and 24 h; Figures 4(a) and 4(b)). In addition, p21 expression was correspondingly increased with increasing concentrations of RVSE (*P* < 0.05 for 300 and 400 *μ*g/mL; Figure 4(c)).

### 3.5. Effect of RVSE on the Bax:Bcl-2 Ratio, and the Levels of Cleaved Caspase-3 and -9 and PARP

**Figure 5:**
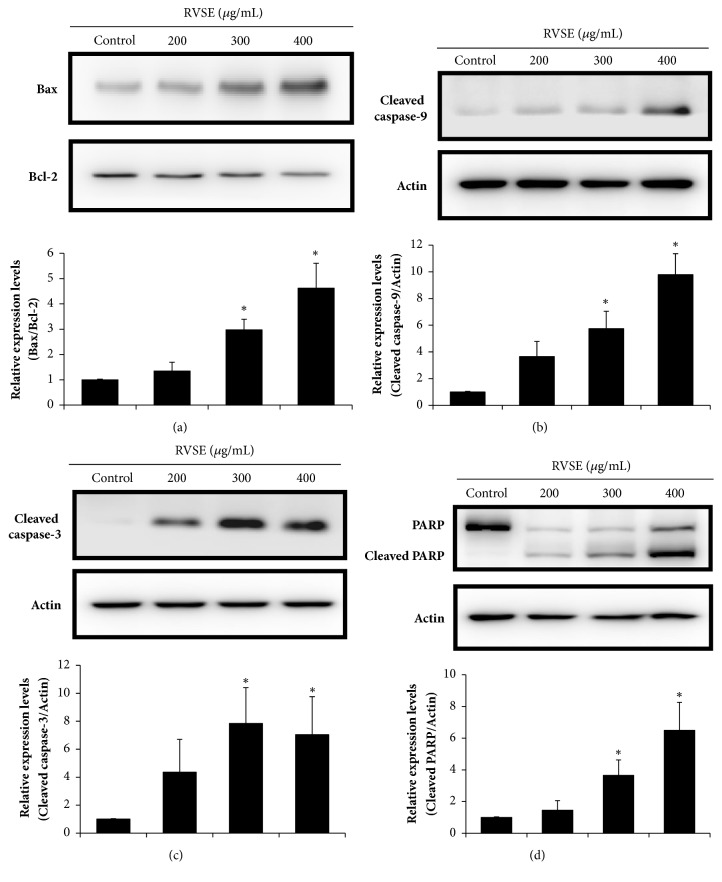
Effects of 200, 300, and 400 *μ*g/mL RVSE for 24 h on the Bax:Bcl-2 ratio, and the cleaved caspase-3 and -9 and PARP levels in MCF-7 cells. Western blotting was performed for the determination of the relative protein levels of (a) Bax:Bcl-2 ratio, (b) cleaved caspase-9, (c) cleaved caspase-3, and (d) PARP and cleaved PARP. *β*-Actin was used as the loading control. A representative of three blots yielding similar results is shown. Relative levels (protein* vs.β*-actin) were evaluated by densitometry. Data are means ± SD of three independent experiments. *∗P* < 0.05, significant difference from the control.

Next, we investigated whether p53-induced apoptosis was associated with mitochondrial membrane potential. The Bax:Bcl-2 ratio was increased by RVSE treatment in a dose-dependent manner (*P *< 0.05 for 300 and 400 *μ*g/mL, Figure 5(a)). We also examined the involvement of caspases and PARP in the induction of apoptosis by RVSE. The cleaved caspase-3 and -9 and PARP levels were evaluated by western blotting (Figures 5(b), 5(c), and 5(d)). The levels of cleaved caspase-3 and -9 and PARP were significantly increased by RVSE treatment (*P *< 0.05 for 300 and 400 *μ*g/mL; Figures 5(b), 5(c), and 5(d)).

## 4. Discussion

*Rhus verniciflua* Stokes is an herbal extract traditionally used to treat various diseases in East Asia and is helpful for some cancer patients [11]. RVSE reportedly has antiproliferative and antiapoptotic effects on various tumor cell lines, e.g., human lymphoma [18], breast cancer [19], osteosarcoma [20], and transformed hepatoma cells [21]. However, the molecular mechanism of the inhibitory effects of RVSE on MCF-7 cells is unclear. Accordingly, we examined the antiproliferative effects of RVSE on MCF-7 cells.

Cancer cells have a high rate of growth due to deregulation of the cell cycle and apoptosis [22]. Therefore, induction of cell cycle arrest is an important target in cancer [23, 24]. Furthermore, RVSE treatment activates AMPK*α*2 in MCF-7 cells and the AMPK pathway may exert a deep influence on RVSE-mediated inhibition of tumor viability [25]. Our findings showed that RVSE significantly inhibited the proliferation of MCF-7 cells in a dose-dependent manner. Following exposure to RVSE, MCF-7 cells in sub-G1 phase accumulated; this effect was significant at 300 and 400 *μ*g/mL RVSE. These results suggest that the RVSE-mediated inhibition of the proliferation of MCF-7 cells is due to induction of apoptosis in sub-G1 phase. Future investigation will focus on explaining the mechanism between apoptosis in sub-G1 phase and tumor cell viability in RVSE-induced signaling pathway.

p53 plays a major role in the response of cells to various stressors by inducing or suppressing the expression of genes related to cell cycle arrest via p21, apoptosis, and DNA repair [26–28]. One of the major mechanisms underlying the effects of p53 is how a cell decides to behave, undergoing either cell cycle arrest via p21 or apoptosis, upon p53 induction [29]. Stresses such as DNA damage, ultraviolet irradiation, hypoxia, oxidative stress, and heat shock induce the expression of two sets of p53-regulated genes. One set regulates the cell cycle and the other apoptosis [30–32]. Our results show that p53 and p21 expression was significantly upregulated by RVSE treatment in a dose-dependent manner, suggesting that p53 and p21 mediate the induction of apoptosis by RVSE.

Caspases are produced as inactive precursors (procaspases); their activation requires proteolytic cleavage [33, 34]. Apoptosis can proceed by activation of the extrinsic death receptor pathway or the intrinsic mitochondrial pathway [35]. The intrinsic mitochondrial pathway involves members of the Bcl-2 family and the activation of caspases (including caspase-3/7 and -9) [36, 37]. In this study, the ratio of Bax (proapoptotic) to Bcl-2 (antiapoptotic) was significantly increased by RVSE treatment in a dose-dependent manner, indicating that RVSE induces apoptosis through the intrinsic mitochondrial pathway. In addition, the levels of cleaved caspase-3 and -9 and PARP were significantly increased by RVSE treatment. Thus, RVSE activates caspase-3 and -9, significantly increasing the cleaved PARP level in MCF-7 cells. Taken together, these findings suggest that the intrinsic mitochondrial apoptotic pathway mediates the anticancer activity of RVSE in MCF-7 breast cancer cells.

RVSE is an aqueous extract from* R*.* verniciflua* Stokes, which has long been used as a food supplement and traditional herbal medicine for various ailments in East Asia [11]. Several studies have evaluated the anticancer effects of* R*.* verniciflua* Stokes [18–21]. Especially, aRVS (allergen-removed RVS) has strong antigrowth and proapoptotic effects on A549 human lung cancer cells and the effects are mediated via the activation of caspases, downregulation of Bcl-2 and Mcl-1, Bax upregulation, p53 hyperphosphorylation, and S6 hypophosphorylation [38]. Other works have focused on the anticancer activity of gallic acid [39–43], a plant polyphenol present in grapes, berries, other fruits, and tea [44, 45]. Gallic acid possesses marked antioxidant [46] and antitumor [47] activity. Furthermore, gallic acid-triggered apoptosis in human colon cancer (HCT116 cells) was associated with the upregulation of the intrinsic p53 signal pathway via the activation of caspases, finally leading to the intrinsic mitochondrial apoptosis pathway [48]. In addition, RVSE contains other phenolic compounds including fisetin, fustin, sulfuretin, and butein [49]. Using HPLC, we identified that only gallic acid is detected in the RVSE. However, fustin and sulfuretin were not detected in the RVSE. Because marker compounds in medicinal herbs can be affected by many factors, including collection time, place, cultivation environment of the plants, temperature, and method of manufacture of the herbal medicine, these analytical data are somewhat different. Therefore, gallic acid may be responsible for the antiproliferative effects of RVSE on MCF-7 breast cancer cells. Further studies in clinical settings are required to promote RVSE as a beneficent candidate for anticancer agent.

## 5. Conclusions

RVSE inhibited the growth of MCF-7 breast cancer cells in a dose-dependent manner by inducing apoptosis in sub-G1 phase via upregulation of p53 and p21. Furthermore, RVSE induced the intrinsic mitochondrial apoptotic pathway, as indicated by an increased Bax:Bcl-2 ratio and elevated cleaved caspase-3 and -9 and PARP levels. Taken together, these findings suggest that the intrinsic mitochondrial apoptotic pathway is involved in the antiproliferative effects of RVSE on MCF-7 breast cancer cells. Thus, RVSE may hold promise for the prevention and treatment of breast cancer. Further investigation should seek to confirm that the anticancer effects of RVSE are recapitulated* in vivo*.

## Data Availability

The data used to support the findings of this study are available from the corresponding author upon request.
